# Magnetic Particle Spectroscopy for Point-of-Care: A Review on Recent Advances

**DOI:** 10.3390/s23094411

**Published:** 2023-04-30

**Authors:** Parsa Yari, Bahareh Rezaei, Clifton Dey, Vinit Kumar Chugh, Naga Venkata Ravi Kumar Veerla, Jian-Ping Wang, Kai Wu

**Affiliations:** 1 Department of Electrical and Computer Engineering, Texas Tech University, Lubbock, TX 79409, USA; 2 Department of Electrical and Computer Engineering, University of Minnesota, Minneapolis, MN 55455, USA

**Keywords:** magnetic particle spectroscopy, biosensor, volumetric assay, surface-based assay, disease detection, point-of-care, food safety

## Abstract

Since its first report in 2006, magnetic particle spectroscopy (MPS)-based biosensors have flourished over the past decade. Currently, MPS are used for a wide range of applications, such as disease diagnosis, foodborne pathogen detection, etc. In this work, different MPS platforms, such as dual-frequency and mono-frequency driving field designs, were reviewed. MPS combined with multi-functional magnetic nanoparticles (MNPs) have been extensively reported as a versatile platform for the detection of a long list of biomarkers. The surface-functionalized MNPs serve as nanoprobes that specifically bind and label target analytes from liquid samples. Herein, an analysis of the theories and mechanisms that underlie different MPS platforms, which enable the implementation of bioassays based on either volume or surface, was carried out. Furthermore, this review draws attention to some significant MPS platform applications in the biomedical and biological fields. In recent years, different kinds of MPS point-of-care (POC) devices have been reported independently by several groups in the world. Due to the high detection sensitivity, simple assay procedures and low cost per run, the MPS POC devices are expected to become more widespread in the future. In addition, the growth of telemedicine and remote monitoring has created a greater demand for POC devices, as patients are able to receive health assessments and obtain results from the comfort of their own homes. At the end of this review, we comment on the opportunities and challenges for POC devices as well as MPS devices regarding the intensely growing demand for rapid, affordable, high-sensitivity and user-friendly devices.

## 1. Introduction

For good reasons, magnetic particle spectroscopy has experienced significant growth in recent years. Unlike other methods, MPS-based bioassays directly measure magnetic responses from either magnetic nanoparticles (MNPs) or magnetic beads (MBs). This direct measurement approach is convenient and efficient (authors note, for convenience, in this review, we use MNP to represent both types of particles), and enables an analysis on minimally processed biological samples, e.g., without filtration, purification and wash steps. In addition, the MNP labels used in MPS-based bioassays bring more advantages to this platform (as well as to most types of magnetic biosensors): (1) the MNPs are superparamagnetic and they do not aggregate in liquid due to zero averaged magnetization in the absence of a magnetic field, which can effectively prevent false magnetic signals and clotting in blood vessels if used for in vivo applications [1]; (2) the magnetic property of MNPs allows for remote control by an external magnetic field, which enables the integration of MPS-based bioassays with drug delivery, hyperthermia, magnetic separation, etc. [2]; (3) the MNPs are very stable even in a harsh environment and will not undergo photobleaching like fluorescent dyes [3]. Furthermore, MNPs made from iron oxides are nontoxic to cells and biological tissues, enabling their current extensive utilization in magnetic hyperthermia therapy, drug delivery, magnetic biosensing, magnetic separation and imaging [4,5,6,7,8].

In this paper, we provided a comprehensive overview of various MPS detection modes, including the single- and dual-frequency driving field-based MPS, and sensing strategies such as surface and volumetric assays. By reviewing the latest research on MPS-based bioassays, the authors suggest that these platforms hold significant promise for diverse biomedical applications due to the distinct magnetic properties and ease of MNPs synthesis and functionalization. Moreover, the use of Brownian relaxation-dominated MNPs for in vivo viscosity and temperature mapping can provide valuable additional features to MPS-based bioassays and magnetic particle imaging (MPI) [9,10,11,12,13]. Due to the speed and simplicity of MPS-based bioassays, they are attracting growing interest as a potential point-of-care (POC) testing tool in various fields, such as medicine, food safety control, agriculture and veterinary medicine [14,15].

**Figure 1:**
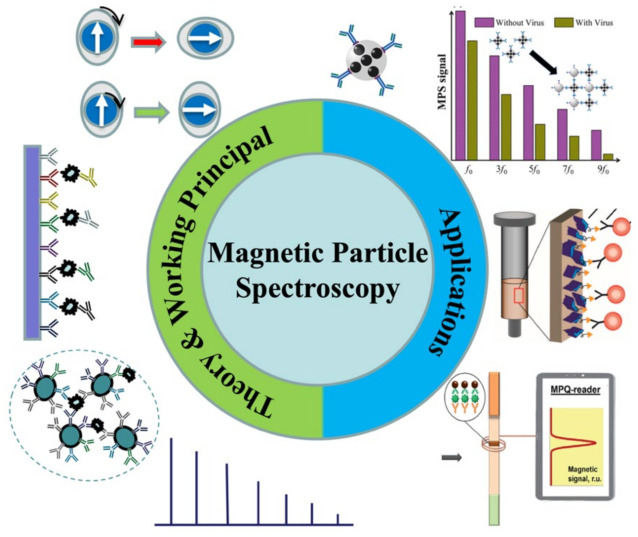
A summary of MPS-based bioassays reviewed in this work. Figure reprinted with permissions from Ref. [16], copyright 2021 American Chemical Society; from Ref. [17], distributed under the terms of the Creative Commons Attribution License; from Ref. [18], copyright 2021 The Royal Society of Chemistry.

This work will provide a systematic review on the mathematical models of MNPs and the MPS-based bioassay mechanisms, as well as present some works on MPS-based biomedical applications, as summarized in Figure 1. Section 2 provides the theories and mechanisms of MPS-based bioassays, the mathematical models of dynamic magnetic responses of MNPs subjected to external magnetic fields, and the Brownian and Néel relaxation time models. In Section 3, we briefly reviewe the MPS portable platforms reported by different groups so far. Section 4 summarizes the utilization of MPS for disease diagnosis, such as for the detection of SARS-CoV-2, Influenza A Virus (IAV), Hepatitis B Virus (HBV), prostate-specific antigen (PSA), etc. In Section 5, applications of the MPS technique in food safety control are reviewed, such as the detection of several toxins such as mycotoxins, aflatoxin, and staphylococcal enterotoxin B (SEB). At the end of this review, we provide peers with some comments on the future challenges and opportunities in POC devices and MPS-based applications.

## 2. Magnetic Particle Spectroscopy (MPS): Mechanisms and Theories

### 2.1. Magnetic Nanoparticles (MNPs)

Magnetic nanoparticles (MNPs) are regarded as highly promising materials with widespread applications in various fields, including magnetic separation, diagnostics, and therapeutics [2,7,19,20,21]. MNPs with proportional sizes to biomolecules have demonstrated outstanding properties, including a high reactivity, significant surface-to-volume ratio and unique magnetic characteristics, compared to their primary bulk materials. In order to produce MNPs, magnetic materials such as pure metals (e.g., Co, Ni, Fe), alloys (e.g., FeCo, alnico, permalloy) and oxides (e.g., Fe_3_O_4_, γ-Fe_2_O_3_, CoFe_2_O_4_) with high saturation magnetizations are preferred. Although pure metals can produce higher saturation magnetizations, they are not suitable for biomedical applications due to their cytotoxicity or susceptibility to oxidation [2]. Iron oxides are currently the most commonly used MNPs due to their highly chemical and colloidal stability, amazing biocompatibility and affordability.

Over the last 30 years, there has been a fascinating era of MNPs synthesis with remarkable physical characteristics for biological and biomedical purposes, and several of these synthesis approaches have been commercially produced [2,19,22,23,24]. For example, various techniques, including ball milling, gas phase condensation (GPC), thermal decomposition, sol–gel and others, have successfully been used to prepare monodispersed magnetic nanoparticles (MNPs) [25,26,27,28]. These synthesis methods offer different kinds of MNPs for a wide variety of applications. Iron oxide MNPs are very popular as they are inexpensive and extremely stable in ambient temperature. Meanwhile, MNPs made from Fe, Co, Ni and their alloys are also of interest due to the raw materials’ high saturation magnetizations. In addition, high-magnetic-moment MNPs (a high magnetic moment per particle) with highly biocompatible, organic or non-organic shells (i.e., the core–shell structure) play a crucial role in the field of nanomedicine [2,27]. For imparting biological recognition and interaction skills, the surface functionalization of MNPs with biomolecules, polymers and ligands is essential.

The magnetic properties of MNPs, such as the magnetic anisotropy and saturation magnetization, are primarily dependent on their crystalline structures, sizes and shapes. The magnetic moment (m) is the product of the magnetic core volume (V_m_) and spontaneous saturation magnetization (M_s_), which is the most significant property of MNPs for nanomedicine-oriented applications [2,29]. For having a higher magnetic signal (in applications such as magnetic biosensing, imaging, etc.) and a stronger magnetic force (in applications such as magnetic manipulation and drug/gene delivery), a higher magnetic moment per MNP is desired. It should be mentioned that the insufficiency of translational crystal symmetry in the surface layer of MNP will inevitably lead to the dissimilarity of the surface layer’s magnetic properties compared to the inner core. As an outcome, lower saturation magnetizations (M_s_) and higher anisotropy constants are observed in MNPs in comparison to their corresponding bulk materials [29,30,31].

### 2.2. Superparamagnetism

Superparamagnetism is a type of unique magnetic property that emerges in small ferro- or ferrimagnetic nanoparticles. When the energy barrier E_b_ is comparable to or lower than the thermal fluctuation energy K_b_T under a finite temperature T, the magnetic moment in an MNP flips direction frequently during a measurement time window τ_m_, resulting in a zero averaged net magnetization, namely the superparamagnetic state. At a specific measurement time and temperature, there is a critical size D_sp_ that determines the transition from a single-domain nanoparticle to superparamagnetic nanoparticle, which varies for different magnetic materials and typically ranges from a few nanometers to several tens of nanometers [32]. Due to the fast flipping of their magnetic moments, superparamagnetic nanoparticles exhibit magnetic moments even in the absence of an external magnetic field. However, when subjected to an external field, their magnetic moments align along the field direction, producing detectable magnetic signals. The magnetic moment of superparamagnetic nanoparticles versus the applied magnetic field is typically a reversible S-shape. In the superparamagnetic state, an external magnetic field can magnetize the nanoparticles similarly to a paramagnet, but with a much larger magnetic susceptibility under small fields [2,33]. For most biomedical applications, the MNPs are generally superparamagnetic in order to avoid aggregation and potential clotting for in vivo applications.

### 2.3. Magnetic Responses of MNPs: The Langevin and Debye Models

In general, in order to analyze the magnetization curves of superparamagnetic nanoparticles (in this work, for convenience, all MNPs mentioned are superparamagnetic), the Langevin function, which is extended by a Debye pre-factor, is used. This model is considered as a dynamic magnetization model of the MNPs. Although the Langevin model is at best valid for slowly alternating magnetic fields or static magnetic fields in which the MNPs have sufficient time to reach an equilibrium state, this model does not define the phase differences between the MNP’s magnetization and the external field [34,35]. The Langevin model of the static magnetization of MNPs subjected to the external magnetic field is expressed as follows (in SI units):

(1) $$M\;\left(\mu_{0}H\right)=M_{S}\cdot L\;\left(\frac{m_{0}\mu_{0}H}{k_{B}T}\right)$$

where $L\left(\xi \right)$, $\xi =\frac{m_{0}\mu_{0}H}{k_{B}T}$, is the Langevin function, $m_{0}$ is the magnetic moment (in $A\cdot m^{2}$) of a single MNP, $M_{S}$ is the saturation magnetization (in A/m) of MNP, H is the externally applied magnetic field (in A/m), $\mu_{0}$ is the vacuum permeability, T is the absolute temperature in Kelvin and $k_{B}$ is the Boltzmann constant ($k_{B}\;\approx 1.38\times 10^{-23}\;J/K$). The Langevin function $L\left(\xi \right)$ is expressed as:

(2) $$L\left(\frac{m_{0}\mu_{0}H}{k_{B}T}\right)=coth\left(\frac{m_{0}\mu_{0}H}{k_{B}T}\right)-\frac{k_{B}T}{m_{0}\mu_{0}H}$$

Generally, MPS and MPI data modeling is introduced by using the simplest nonlinear model available: an ensemble of noninteracting MNPs’ quasi-static magnetization (as a function of the applied magnetic field H) can be explained by the Langevin function *L*(ξ), where $\xi =\frac{m_{0}\mu_{0}H}{k_{B}T}=\frac{E_{m}}{E_{T}}$ suggests that the ratio of magnetostatic energy $E_{m}=m_{0}\mu_{0}H$ and thermal energy $E_{T}=k_{B}T$ [36,37].

As mentioned previously, the Langevin model cannot provide the phase information of MNPs subjected to fast-changing magnetic fields. At low frequencies of the driving magnetic field, the magnetic moment of the MNP can closely track the driving field, and the magnetic susceptibility $\chi_{0}$ is a real number. However, as the frequency of the driving field increases, a phase lag φ arises between the magnetic moment of the MNP and the driving magnetic field, resulting in a complex magnetic susceptibility that is described by the Debye model [34,35]:

(3) $$\varphi =\tan^{-1}\left(\omega t\right)$$

(4) $$\chi |\left(\omega \right)=\frac{\chi_{0}}{1+j\omega \tau \;}=\frac{\chi_{0}}{\sqrt{1+{\left(\omega \tau \right)}^{2}}}e^{-j\tan^{-1}\left(\omega \tau \right)}=\;\left|\chi \right|e^{j\varphi }\;$$

where $\chi_{0}$ is the static magnetic susceptibility, *ω* is the angular frequency and *τ* is the adequate relaxation time of the MNP. It is worth mentioning that the Debye model is unequivocally applicable to small fields.

### 2.4. Brownian and Néel Relaxation Models

**Figure 2:**
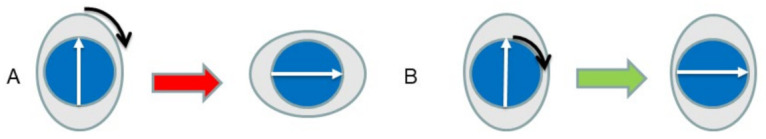
(**A**) Brownian relaxation: the entire particle including its magnetic moment *m* physically rotates in the fluid. (**B**) Néel relaxation: magnetic moment rotates within stational particle core. White arrows represent the magnetization directions in the MNPs. Black arrows represent the rotational directions.

The MNP’s dynamic magnetizations and the rotational freedom hold an important role in MPS-based applications (especially in volumetric-based MPS bioassays) [38]. Thus, a dynamic magnetization model is required to predict the physical behavior of MNPs for assays. There will be a reorientation of the magnetic moments of the MNPs subjected to externally applied magnetic fields. As shown in Figure 2, the physical rotation of the MNP’s magnetic moment along with its hydrodynamic shell is called the Brownian rotation (Figure 2A) and the internal rotation of the magnetic moment inside the stational MNP is called Néel relaxation (Figure 2B). These two distinct physical processes are responsible for the effective relaxation behavior that we observe. Both relaxations can be understood as procedures of magnetic decay with certain relaxation time constants. In order to minimize magnetostatic energy, which is countered by the thermal fluctuations (*k_B_T*), the magnetic moments of MNPs suspended in liquid follow the external magnetic field through the joint Brownian and Néel relaxation processes. These two procedures cooperatively affect the dynamic magnetic responses of MNPs to the drive fields. Nonetheless, it should be marked that, generally, Brownian and Néel relaxation are coupled processes and cannot be considered as separable.

The rotational diffusion coefficient of a spherical MNP follows the Stokes-Einstein-Debye equation [39,40,41]:

(5) $$D_{r,B}=\frac{E_{T}}{\xi }=\frac{k_{B}T}{6\eta V_{h}}$$

where *η* is the dynamic viscosity of the liquid media, $V_{h}=\frac{\pi d_{h}^{3}}{6}$ is the hydrodynamic volume of the MNP and *d_h_* is the hydrodynamic diameter. The zero-field Brownian relaxation time is given by:

(6) $$\tau_{B0}=\frac{1}{2D_{r,B}}=\frac{3\eta V_{h}}{k_{B}T}\;\;$$

The dynamic viscosity $\eta =\eta \left(T\right)$ significantly decreases with an increasing ambient temperature, which makes the Brownian relaxation time dependent on temperature. This relationship can be explained by the Arrhenius-Andrade equation [9]:

(7) $$\eta \left(T\right)=\eta_{0}\cdot exp\left(\frac{E_{a}}{k_{B}T}\right),$$

where $\eta_{0}$ is the viscosity of the fluid at a reference temperature (usually taken as room temperature) and $E_{a}$ is the activation energy.

As shown in Equation (8), the effective anisotropy constant K (which includes the crystal and shape anisotropies), the temperature T and the magnetic core volume $V_{C}$ are the parameters on which the characteristic zero-field Néel relaxation time $\tau_{N0}$ is exponentially dependent on. The argument of the exponential function $KV_{c}/k_{B}T$ describes the ratio of anisotropy energy and thermal energy. It is scaled with *τ*_0_ (typically around 1 ns), which is also dependent on the material-specific Gilbert damping parameter α and the gyromagnetic ratio γ. The $\tau_{0}^{*}$ is a characteristic time scale for the relaxation of the magnetization in a magnetic material to its equilibrium value in the absence of an external magnetic field. The zero-field Néel relaxation time $\tau_{N0}$ is modeled as below [42]:

(8) $$\left.\begin{array}{c} \tau_{N0}=\frac{1}{2D_{r,N}}=\frac{\sqrt{\pi }}{2\sqrt{\frac{KV_{C}}{k_{B}T}}}\frac{M_{S}\left(1+\alpha^{2}\right)}{2K\gamma \alpha }exp\left(\frac{KV_{C}}{k_{B}T}\right)\\ \tau_{0}=\frac{M_{S}\left(1+\alpha^{2}\right)}{2K\gamma \alpha }\\ \tau_{0}^{*}=\frac{\sqrt{\pi }}{2\sqrt{\frac{KV_{c}}{k_{B}T}}}\frac{M_{S}\left(1+\alpha^{2}\right)}{2K\gamma \alpha } \end{array}\right\}$$

The calculation of the effective relaxation time involves considering the superposition of both relaxation processes as they are time constants of two different mechanisms of magnetic decays. Therefore, the effective relaxation time is typically modeled as a parallel arrangement of both zero-field relaxation processes, where the faster process predominates, and the effective zero-field relaxation time is described as [43]:

(9) $$\tau_{eff0}=\frac{\tau_{B0}.\tau_{N0}}{\tau_{B0}+\tau_{N0}}$$

It should be noted that the abovementioned equation is only valid for low-frequency and small-magnitude fields, which is usually not the case for MPS and MPI applications. One can well imagine that a high magnetic field strength shortens the relaxation time because of the large magnetic torque acting on an MNP. There is no absolute relaxation during dynamic excitation since the particles are dragged continuously. Nevertheless, the dynamic magnetic behaviors of MNPs can be modeled by field-strength-dependent relaxation times. Yoshida and Enpuku [44] solved the Fokker-Planck equation for the non-zero field Brownian relaxation time model of MNPs below [45]:

(10) $$\tau_{BH}=\frac{\tau_{B0}}{\sqrt{1+0.126\;\xi^{1.72}}}$$

The non-zero field Néel relaxation time model is expressed as [46]:

(11) $$\tau_{N}=\frac{\tau_{N0}}{\sigma_{eff}\left(1-h^{2}\right)}={\left(\frac{\sqrt{\frac{\sigma_{eff}}{\pi }}}{1+\frac{1}{\sigma_{eff}}}+2^{-\sigma -1}\right)}^{-1}\times {\left(\frac{1-h}{e^{\sigma_{eff}\left(1-h^{2}\right)}-1}+\frac{1+h}{e^{\sigma_{eff}\left(1+h^{2}\right)}-1}\right)}^{-1},$$

where $\sigma_{eff}$ is the anisotropy parameter and *h* is the strength parameter for an external field strength *H*.

### 2.5. Higher Harmonics of MNPs Subjected to Sinusoidal Magnetic Fields

Due to the nonlinear magnetic responses of MNPs subjected to external excitation magnetic field *H*(*t*), the induced magnetic responses, *M*(*t*), contain not only the ‘modulation field’ frequency *f* but also a series of higher odd harmonics such as 3*f*, 5*f*, 7*f*, and 9*f*, etc. (in a mono-frequency driving field scenario). Appropriate filtering was used to extract these higher harmonics for analysis. In MPI, a magnetic gradient field that is equal to zero in the FFP (field free point) and increases toward the edges is applied on top of the ‘modulation field’ in order to suppress these harmonics for spatial encoding purposes. The magnetic responses in the form of harmonics from the MNPs outside the FFP are fully saturated by this non-zero gradient field, and those harmonics are largely suppressed. In comparison to the odd harmonics generated by MNPs within the FFP, the amplitudes of these harmonics from outside the FFP are insignificant. Therefore, MNPs within the FFP are the only magnetic signal sources responsible for 3D tomographic imaging in MPI [47,48]. MPI emerges as a new 3D imaging technique for real-time in vivo scanning, and it is expected to reach the clinical stage soon [49].

Meanwhile, various MPS platforms, which originated from MPI, have been described for use in bioassays and have subsequently become a novel research focus in the field of magnetic bioassays [48,50,51]. Nikiet et al. [50] and Krause et al. [51] independently reported the first-generation MPS platforms. In 2006, a magnetic bioassay platform was developed that utilized a magnetic drive field with two frequency components (*f*_H_ and *f*_L_) to drive MNPs into the saturation region. Subsequently, another version of the MPS platform for bioassay applications was introduced that only used a magnetic drive field with a single-frequency component *f* [9,42].

It should be noted that the modulation field in MPI and the magnetic drive field in MPS are both sinusoidal fields that are utilized to repeatedly saturate MNPs. However, in order to differentiate between these two techniques, the term “magnetic drive field” is used only in MPS. In MPS, the magnetic drive field is responsible for triggering the nonlinear magnetic responses of MNPs and higher harmonics that serve as indicators for bioassay applications. Additionally, since MPS does not require tomographic scanning, the gradient field can be removed.

**Figure 3:**
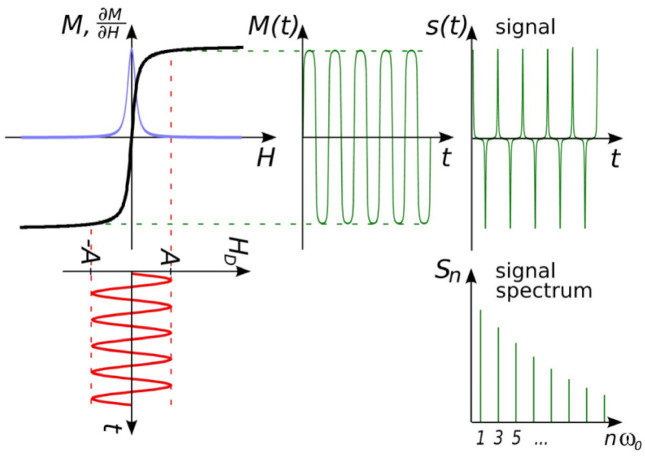
MPS platform basic concept idea. The response is determined by the magnetization of MNPs (*M*), the magnetic field strength (*H*), the magnetic field strength of the right field (*H_D_*), and the passage of time (*t*). Figure reprinted from Ref. [61], distributed under the terms of the Creative Commons Attribution License.

Herein, different MPS platforms are classified by the formats of magnetic drive fields. The first one is the mono-frequency drive field platform, where there is only one sinusoidal drive field expressed as $H\left(t\right)=A\sin\left(2\pi ft\right)$. The magnitude of this drive field is large enough to repeatedly saturate MNPs. As a result, the nonlinear magnetic responses lead to higher harmonics of 3*f* (the third harmonic), 5*f* (the fifth harmonic), 7*f* (the seventh harmonic), …, which are generated by MNPs, filtered and collected by pick-up coils as information. This is similar to the scenario of MNPs within the FFP in MPI as shown in Figure 3.

The second type of MPS platform is the dual-frequency drive field design, where there are two sinusoidal magnetic drive fields that are applied. A drive field with a large amplitude and low frequency, expressed as $H_{L}=A_{L}\sin\left(2\pi f_{L}t\right)$ is applied to repeatedly saturate MNPs, while there is a second drive field with a high frequency and low amplitude, expressed as $\;H_{H}\left(t\right)=A_{H}\sin\left(2\pi f_{H}t\right)$, that is applied to modulate the higher harmonics to a high-frequency region. Due to the nonlinear dynamic magnetic responses of MNPs, higher harmonics at frequencies of $f_{H}\pm 2f_{L}$ (the third harmonics), $f_{H}\pm 4f_{L}$ (the fifth harmonics), $f_{H}\pm 6f_{L}$ (the seventh harmonics), …, $f_{L},3f_{L},5f_{L},$ …, and $f_{H},3f_{H},5f_{H}$, …, are observed. From a macro perspective, both MPS platforms record the higher odd harmonics from nonlinear dynamic magnetic responses of MNPs aside from the magnetic drive fields [50,51,52,53]. The higher harmonics amplitudes are comparable to the number of MNPs, drive fields (including field amplitude and frequency) and magnetic moment per particle, and are inversely proportional to the phase lag of magnetic moments to the drive fields [9,52,53,54,55]. The Brownian and Néel relaxation processes can be influenced by several factors, including but not limited to the temperature, magnetic anisotropy, viscosity of the liquid medium, size of the magnetic core, hydrodynamic size and saturation magnetization of the MNPs. The phase lag *φ* directly relates to the effective relaxation time of MNPs (by the joint effects of Brownian and Néel relaxation processes) [42,52,56]. As a result, the higher harmonics are also reported as metrics used to measure the temperature (*T*) and viscosity (*η*) of Mthe NP medium, saturation magnetization (*M_s_*), hydrodynamic size (*V_h_*) and magnetic core size (*V*_c_) of MNPs [57,58,59,60].

### 2.6. Volumetric and Surface MPS Bioassays Mechanisms

Currently, two types of MPS bioassay strategies are investigated frequently: surface- and volumetric-based methods. The innate main differences between these two methods are that the volumetric-based bioassay monitors the bound status (or, the rotational freedom) of MNPs in the presence of target analytes whereas the surface-based bioassay method monitors the amount of MNPs captured onto a nonmagnetic reaction substrate in the presence of target analytes.

**Figure 4:**
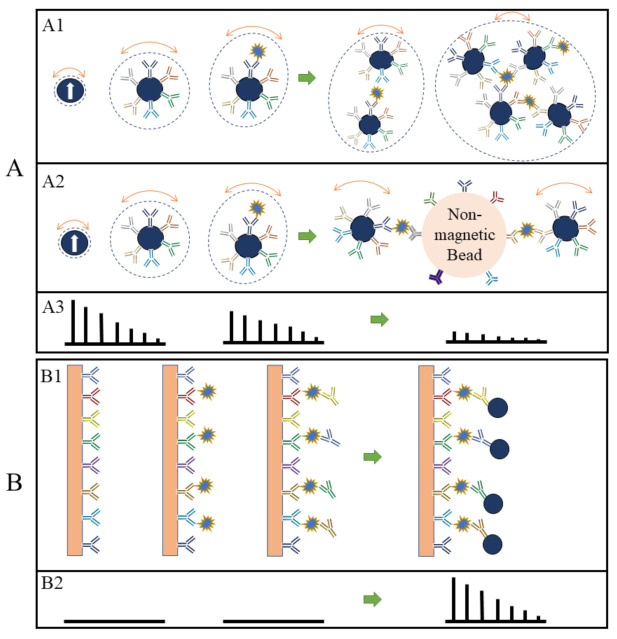
(**A**,**B**) depict the volumetric- and surface-based MPS bioassay mechanisms, respectively. (**A1**,**A2**,**B1**) schematically show the different bioassay steps. (**A3**,**B2**) show the corresponding MPS spectra observed at each bioassay stage.

In the volumetric-based MPS bioassay method, MNPs are functionalized with capture probes that can specifically bind to target analytes (for example, via antibody–antigen recognition) in liquid. As shown in Figure 4A, MNPs are surface-functionalized with polyclonal detection antibodies. In the presence of target antigens, these polyclonal antibodies will bind to different epitopes from each protein molecule, thus causing the cross-linking of MNPs. As a result, the hydrodynamic sizes of MNPs increase, as well as the Brownian relaxation time. Thus, lower harmonic amplitudes and a larger phase lag are observed as schematically drawn in Figure 4(A3) [48,62,63]. In another example of the volumetric-based method as shown in Figure 4(A2), non-magnetic beads are introduced as the reaction surface to further reduce the rotational freedom of the MNPs. As a result of these binding and clustering events, the MPS spectrum in Figure 4(A3) becomes weaker and weaker as Brownian relaxation is hindered. The volumetric-based MPS bioassay method is a homogeneous bioassay platform that spots the target analytes straight from the liquid sample without wash steps, making it suitable for future point-of-care (POC) applications. Currently, the volumetric-based MPS bioassays demonstrate a high bioassay sensitivity and specificity, as well as the ability to multiplex different analytes in a single sample [64,65,66].

On the other hand, in the surface-based MPS assay method, the MNPs are captured and fixed onto a reaction surface via a specific binding process in the presence of target analytes. Their magnetic moments following the AC magnetic field through the Néel relaxation process are recorded and higher harmonics are extracted for analysis. This surface-based MPS bioassay method is similar to the traditional surface biosensors such as lateral flow (LF) tests [67,68,69,70,71], surface-enhanced Raman spectroscopy (SERS) biosensors [72,73,74,75,76], giant magnetoresistive (GMR) biosensors, etc. [66,77,78,79,80,81]. These sorts of biosensors come with a chemically utilized reaction surface to capture target analytes and then label them (these could be magnetic or fluorescent labels), where these labels are bound to the reaction surface through specific validation (such as antibody–antigen, DNA–DNA, etc.). The magnetic or optical signals are used to detect the target analytes, as illustrated in Figure 4(B1) using a sandwich bioassay design as an example. The biofluid sample is passed over the reaction surface, where the target analytes are captured through antibody–antigen-specific binding. The molecules and compounds that are not bound to the reaction surface are removed by washing. After that, MNPs are added, which bind to one end of detection antibodies. The extra unbound MNPs are washed out, leaving captured MNPs on the substrate. The number of MNPs captured on the substrate is related to the number of target analytes present in the testing sample, and the higher harmonics’ amplitudes are proportional to the remaining MNPs. Figure 4(B2) shows the MPS spectra before and after the capture of MNPs in the presence of target analytes. The biological matrix does not produce any significant harmonic signals since biological tissues and fluids are nonmagnetic or paramagnetic. The only magnetic signal sources responsible for MPS spectra are the MNPs that are captured and fixed on the substrate [52].

## 3. MPS Platforms

**Figure 5:**
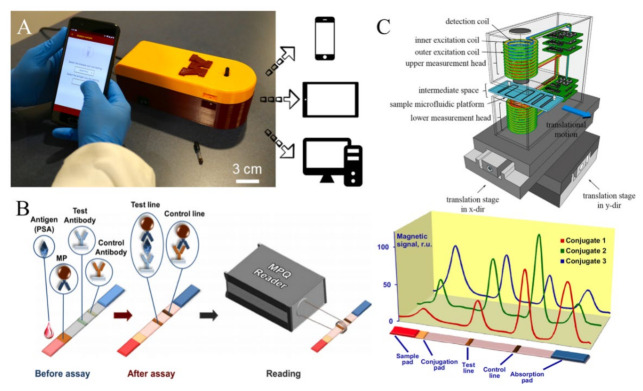
(**A**) Photograph of the MagiCoil POC device with a smartphone application, reported by Jian-Ping Wang’s lab. The overall dimensions of the device are 212 mm (L) X 84 mm (W) X 72 mm (H). (**B**) The MPQ POC device combined with lateral flow assay strip, reported by Petr I. Nikitin’s lab. (**C**) The p-FMMD platform combined with microfluidic channels for fully automatic bioassays, reported by Hans Joachim Krause’s lab. (**A**) reprinted with permission from Ref. [62], copyright 2021 American Chemical Society. (**B**) reprinted with permission from Ref. [64], copyright 2015 Elsevier B.V. (**C**) reprinted with permission from Ref. [84], copyright 2016 Elsevier B.V.

In areas with limited access to medical resources, a portable, quantitative bioassay device will be useful and reduce the load on local healthcare systems. Over a decade’s development, several groups around the globe have reported MPS point-of-care (POC) devices that are readily used for on-site disease diagnosis. For example, a team from the University of Minnesota reported a MagiCoil POC device as shown in Figure 5A [62]. It is a volumetric-based MPS bioassay platform. This MagiCoil POC device provides a completely automated, one-step, wash-free assay along with a user-friendly smartphone interface. This platform is quite versatile: by simply changing the surface functionalization on MNPs, it allows for the detection of different diseases. Although MPS-based bioassays exhibit great sensitivities, as described in several literature, this POC device currently struggles with insufficient sensitivity and requires more enhancements. It is anticipated that this type of portable device would allow for one-step field testing in nonclinical settings such as classrooms, residences and offices, as well as laboratory-based bioassays [62,82].

The Petr I. Nikitin’s lab reported a magnetic particle quantification (MPQ) POC device as shown in Figure 5B [64]. This platform combined the MPS technique and lateral flow assay (LFA) by replacing the Au nanoparticle labels with MNPs. Here, a surface-based MPS assay mechanism was applied. The capture-probe-functionalized lateral flow membrane provides a large reaction surface for the specific binding of ‘MNP–detection probe–target analyte’ complexes. This wash-free assay strategy first preloads ‘MNP–detection probe’ complexes at the conjugation pad. Then, a biofluid sample containing target analytes is dropped on the sample pad. Due to the capillary effect, the biofluid flows toward the absorption pad, bringing the ‘MNP–detection probe–target analyte’ through the test line and control line. The test line is functionalized with capture probes that can specifically bind to the target analytes and fix the MNP labels to the surface of the test line, while the control line is functionalized with probes that capture the unbound ‘MNP–detection probe’ complexes. This control line serves as an indicator of the working status of the test strip in order to avoid false negative scenarios.

The Hans Joachim Krause’s lab combined microfluidic channels with an MPS platform, named a planar-frequency mixing magnetic detection (p-FMMD) device, as shown in Figure 5C [83,84]. This makes the surface-based MPS bioassay fully automatic, freeing the end users’ hands while maintaining the advantage of a high detection sensitivity in this type of assay mechanism. They demonstrated the feasibility of detecting amyloid beta 42 (Aβ_42_), a promising biomarker of Alzheimer’s disease, with an LOD of 23.8 pg/mL. The deployment of this system demonstrates the possibility of fully automatic, fast and portable disease screening.

## 4. MPS-Based Disease Diagnosis

### 4.1. MPS for SARS-CoV-2 Detection

MPS platforms have been frequently reported for the diagnosis of many diseases. One most recent example was the epidemic of respiratory syndrome coronavirus 2 (SARS-CoV-2), which threatened global medical systems and economies and ruled our daily living. A patient who has been infected with SARS-CoV-2 may suffer from severe pneumonia and acute respiratory distress syndrome. The first SARS-CoV-2 infection case was reported in December 2019, and then spread across the entire world, which caused severe problems in medical systems, trading, economics and other social and intercontinental issues. Up until early February 2023, the World Health Organization (WHO) reported more than 750 million cases of infection and 6.8 million deaths in the world. In addition to control measures such as social distancing and washing protocols, WHO strongly recommended a large amount of testing for both symptomatic persons and their contacts. Controlling the outbreak of this disease had become one of the most critical and crucial strategies throughout the entire world. Therefore, a prompt, low-cost, rapid and sensitive diagnosis was the most important measure for controlling the outbreak of SARS-CoV-2 and any future disease outbreaks [16,85]. Currently, polymerase chain reaction (PCR) tests are considered as the standard diagnostic method for SARS-CoV-2 infection due to their high sensitivity and specificity. However, these tests involve several complicated experimental procedures that can take a few hours to generate results. Additionally, the required instrumentation and expertise are costly and not readily available in developing and underdeveloped countries. Therefore, there is an urgent need to explore new cost-effective methods and instrumentation for the prompt and accurate diagnosis of SARS-CoV-2 infection [16].

**Figure 6:**
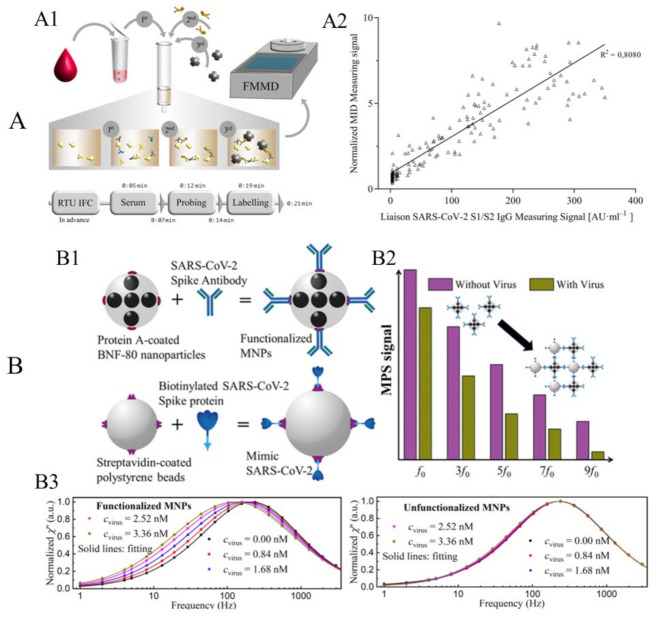
(**A**) Example of surface-based MPS bioassay for the detection of SARS-CoV-2-specific antibodies. (**A1**) Schematic workflow of serological magnetic immunodetection for detection of SARS-CoV-2-specific antibodies in human serum using the surface-based MPS bioassay strategy. (**A2**) Correlation of measured signals by MPS and the certified DiaSorin Liaison SARS-CoV-2 S1/S2 IgG assay. (**B**) Example of volumetric-based MPS bioassay for the detection of mimic SARS-CoV-2 virus. (**B1**) Schematic of functionalized MNPs and mimic SARS-CoV-2 virus particles. (**B2**) Schematic of the harmonic spectra of functionalized MNPs with and without the addition of mimic virus. (**B3**) Experimental results of normalized imaginary parts χ″ of functionalized and unfunctionalized MNPs mixed with different mimic virus concentrations. (**A**) reprinted from Ref. [86], distributed under the terms of the Creative Commons Attribution License. (**B**) reprinted with permission from Ref. [16], copyright 2021 American Chemical Society.

Pietschmann et al. applied a surface-based MPS bioassay strategy for the detection of SARS-CoV-2-specific IgG from patient sera [86]. As shown in Figure 6(A1), the method involves the use of immunofiltration columns (RTU IFC) coated with S1 protein and blocked for ready-to-use serum application. Serum passes through the column by gravity flow and SARS-CoV-2-specific antibodies are enriched within the matrix. After washing, a biotinylated human IgG-specific secondary antibody is applied, followed by streptavidin-functionalized MNPs that rapidly and strongly interact with biotin. Finally, MNPs are quantified using their FMMD portable MPS device, and the harmonics generated by captured MNPs should be proportional to the number of SARS-CoV-2 antibodies retained within the matrix. As shown in Figure 6(A2), a good correlation between the MPS-based assay and the Liaison-based assay (DiaSorin, Italy) was observed. Especially in the range up to 150 AU, a strong correlation is observed, and an R^2^ = 0.81 indicates a comparable assay outcome between Liaison- and MPS-based assays. They reported that each MPS assay takes 21 min, and a sensitivity of 97% and a specificity of 92% are achieved based on the analysis of 170 sera from hospitalized patients.

Jin Zhong et al. reported a volumetric-based MPS assay scheme for the detection of mimic SARS-CoV-2 virus particles, namely the SARS-CoV-2-spike-protein-coated polystyrene beads [16]. They used protein-A-coated multi-core 80 nm BNF-80 MNPs (purchased from micromod Partikeltechnologie GmbH, Rostock, Germany) with an iron concentration of 5.5 mg/mL and a molar particle concentration of 20 pmole/mL. These MNPs are anchored with anti-SARS-CoV-2 spike protein antibodies (purchased from Biomol GmbH, Hamburg, Germany), as illustrated in Figure 6(B1). Because of the strong binding affinity between protein A and the Fc fragment of the antibody, the anti-SARS-CoV-2 spike protein antibodies can readily attach to the surface MNPs after 12 h of incubation at 4 °C without any additional wash steps. Then, these antibody-functionalized MNPs are mixed with different concentrations of SARS-CoV-2-spike-protein-coated polystyrene beads (i.e., the mimic SARS-CoV-2 virus particle). As shown in Figure 6(B2), the higher harmonic spectra are influenced by the addition of mimic SARS-CoV-2 particles since these BNF-80 MNPs are Brownian-relaxation-dominated. In the absence of SARS-CoV-2 mimic particles, the functionalized MNPs can rotate freely, following tightly with the external driving fields, producing a strong harmonic signal. However, in the presence of SARS-CoV-2 mimic particles, MNPs bind to the mimic SARS-CoV-2 and their effective hydrodynamic sizes increase. As a result, the lower degree of rotational freedom leads to a significant decrease in the MPS spectra. Consequently, the amount of SARS-CoV-2 mimic particles present in the liquid sample can be determined by analyzing the MPS spectra. Their proposed approach allows for the rapid detection of mimic SARS-CoV-2 with a limit of detection of 0.084 nM (5.9 fmole) for mimic virus particles. This approach has great potential for designing an affordable POC device for fast and sensitive diagnostics of SARS-CoV-2.

They further investigated the effect of the binding status on the AC magnetic susceptibility (ACS) of MNPs. The imaginary parts χ″ of the ACS for functionalized and unfunctionalized MNPs are shown in Figure 6(B3). For functionalized MNPs, the peak frequency shifts to a lower frequency as the concentration of the mimic virus increases, indicating an increase in the effective Brownian relaxation time τ_B_. In contrast, the χ″ for unfunctionalized MNPs does not show a significant shift when increasing the mimic virus concentration. The changes in the χ″ of functionalized MNPs are more significant than those of unfunctionalized MNPs, suggesting that specific binding behaviors between the mimic SARS-CoV-2 virus and functionalized MNPs play a dominant role. In the case of unfunctionalized MNPs, τ_B_ increases by approximately 1.05 times (from 0.687 to 0.719 ms), whereas, for functionalized MNPs, τ_B_ increases significantly by a factor of approximately 1.74 (from 0.742 to 1.292 ms).

### 4.2. MPS for Other Disease Diagnosis

Besides the SARS-CoV-2 detection, MPS has also been reported for the diagnosis of other diseases. For example, using the anti-thrombin DNA aptamer-functionalized MNPs, Zhang et al. reported the successful detection of thrombin on a volumetric-based MPS assay platform [87]. The higher harmonics are detected from nanogram quantities of iron within 5 s on their lab’s MPS benchtop system (called MSB). Using a streptavidin–biotin binding system, they achieved a detection limit of lower than 150 pM (0.075 pmole), making it much more sensitive than previously reported techniques based on MNP detection. Like the clustering model introduced in Figure 4(A1), the authors functionalized two types of anti-thrombin DNA aptamers (15 mer aptamer and 29 mer aptamer) onto two groups of MNPs individually. The binding of DNA aptamers to target thrombin causes the crosslinking of two groups of MNPs and leads to a noticeable change in harmonic spectra. With this method, the researchers showed that thrombin can be detected with a high sensitivity (4 nM or 2 pmole). Since the harmonic signal is also proportional to the amount of MNPs from the liquid sample (especially for volumetric-based MPS assays, where unbound MNPs are not removed), in order to get rid of the MNP amount, which causes variations between different samples, the authors proposed using harmonic ratios (such as the amplitude ratio of the fifth over the third harmonics, R35) as MNP-concentration-independent indicators of the binding status and target analyte concentrations.

**Figure 7:**
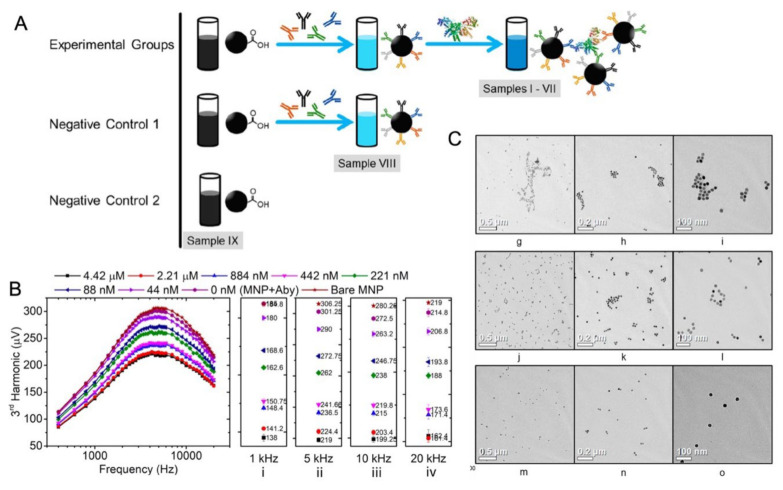
Volumetric-based MPS platform with a dual-frequency drive field used to detect H1N1 nucleoprotein. (**A**) The experimental and negative control groups consist of MNP–antibody complexes with different concentrations of H1N1 nucleoprotein (indexes I–VII), MNP–antibody complex without H1N1 nucleoprotein (index VIII) and a bare MNP suspension (index IX). (**B**) The 3rd harmonics of MNPs were taken from samples I–IX at magnetic drive field frequencies ranging from 400 Hz to 20 kHz. (i–iv) show the 3rd harmonic amplitudes measured at 1 kHz, 5 kHz, 10 kHz and 20 kHz, respectively. (**C**) The bright-field TEM images of MNPs illustrate the different degrees of MNP clustering in the presence of H1N1 nucleoprotein: (g)–(i), (j)–(l), and (m)–(o) represent the TEM images for samples labeled as 2.21 μM, 88 nM, and Bare MNP in (B), respectively. Figure reprinted with permission from Ref. [88], copyright 2020 American Chemical Society.

In another work, Wu et al. reported a volumetric-based MPS method for the detection of the H1N1 nucleoprotein (Catalog# 11675-V08B, Sino Biological Inc., Beijing, China) [88]. As shown in Figure 7A, the rabbit IgG polyclonal antibody (Catalog# 11675-T62, Sino Biological Inc., Beijing, China) is anchored onto the MNPs through a standard EDC (carbodiimide) cross-linking reaction scheme. In the experimental groups, cross-linking takes place between the antibody-functionalized MNPs and the H1N1 nucleoprotein, which leads to the MNP clusters. In the negative control groups, either MNPs are not functionalized with antibodies (labeled as ‘Negative Control 2′ and ‘Bare MNP’) or functionalized MNPs are not mixed with the target H1N1 nucleoprotein (labeled as ‘Negative Control 1′). The third and the fifth harmonics, along with the third over the fifth harmonic ratios (R35), were used as metrics for the quantification of target analytes from bio-fluids. These harmonics from nanogram quantities of iron oxide MNPs can be detected within 10s. Figure 7B shows the third harmonic amplitudes of MNPs added with different concentrations of the H1N1 nucleoprotein compared with the negative control groups (i.e., 0 nM and bare MNP). They observed that the H1N1 nucleoprotein can be detected with a high sensitivity (as low as 44 nM or 4.4 pmole) using this volumetric detection scheme, which is comparable to the analytical sensitivity of fluorescent assays. The transmission electron microscopy (TEM) images of MNPs after bioassays were taken. As shown in Figure 7C, higher concentrations of target analytes lead to higher degrees of MNP clusters, whereas, for the control group, MNPs are well-dispersed, and no significant clusters are observed, confirming that the MPS harmonic signal change is caused by the MNP clusters and the lower degree of rotational freedom. By combining MPS with the artificially induced MNP clusters, they achieved a sensitive, rapid and wash-free magnetic bioassay. Furthermore, this detection strategy is suitable for targeting and quantifying a wide range of biomarkers.

**Figure 8:**
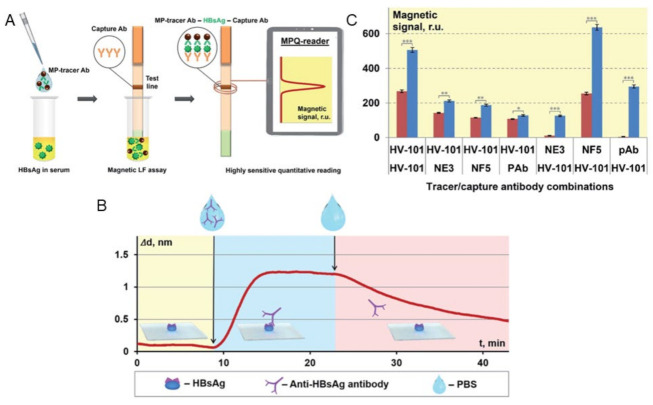
MPS surface-based assay utilizing LF technique for the identification of polyvalent HBsAg. (**A**) Principle of MPS combined with LF assay to identify polyvalent HBsAg. (**B**) The interaction between monoclonal antibody HV-101 and HBsAg immobilized on the sensor chip’s surface is depicted in the sensogram characterization. (**C**) Using different combinations of detection and capture antibodies, the magnetic signals at the test line were measured in response to positive (10 ng/mL of HBsAg) and negative (no HBsAg) serum samples. *p* values (*, **, and *** indicate *p* < 0.05, *p* < 0.01, and *p* < 0.001, respectively) Figure reprinted with permission from Ref. [18], copyright 2021 The Royal Society of Chemistry.

Bargina et al. proposed a surface-based MPS assay strategy that utilizes lateral flow (LF) strips and MNP labels in a sandwich configuration to detect a polyvalent hepatitis B surface antigen (HBsAg) [18]. In order to conduct the test, a serum sample is mixed with MNPs that are conjugated with anti-HBsAg antibodies (in their work, both monoclonal (mAb) and polyclonal (pAb) anti-HBsAg antibodies were tested). Liquid samples are prepared by spiking different amounts of HBsAg in healthy human serum. The serum sample is first mixed with functionalized MNPs for specific binding in the liquid phase as shown in Figure 8A. Through capillary forces, the sample moves toward the absorbent pad and flows onto the test line of the LF strip, where capture antibodies that recognize another available epitope of HBsAg are immobilized. If HBsAg is present in the serum sample, the sandwich structure immobilizes MNPs at the test line (Figure 8A) in order to analyze the binding kinetics and affinity of four mono- and polyclonal antibodies (mAb clones HV-101, NE3, NF5 and pAb) to the HBsAg. The sensograms were collected to depict the real-time association and dissociation between HBsAg and the antibodies using optical label-free biosensors known as SCI and SPI. An example of a characteristic sensogram of interaction between mAb HV-101 and HBsAg is shown in Figure 8B.

It is worth noting that, when detecting a polyvalent antigen with multiple antibody binding sites in a sandwich-type assay, a single antibody can be used as both the detection and capture antibody. In order to investigate the effectiveness of this approach, the researchers compared the performance of mAb clone HV-101, which had optimal kinetic characteristics, when used in combination with other antibodies (mAb clones NE3, NF5 and pAb) or when used simultaneously as both the detection and capture antibody. The magnetic signal increment (ΔS) was then calculated as the difference between signals obtained from the test line with HBsAg-positive and HBsAg-negative serum samples as shown in Figure 8C. They also used the MPS platform to find the ideal quantity of conjugated detection antibodies on MNPs, where different amounts of pAb (4.4, 8.8, 35.5 and 71 μg) were conjugated to 300 μg MNPs, respectively. These MNP-pAb conjugates were then applied for detecting negative (no HBsAg) and positive (1 and 10 ng/mL HBsAg) serum samples. They reported that a pAb amount of 35.5 μg conjugated to 300 μg MNPs is optimal for an efficient interaction with the polyvalent HBsAg since it provides a sufficiently high MPS signal in positive samples and low non-specific signals in negative ones.

Another interesting application of MPS is the characterization of inflammation and infection. Inflammation is a natural defensive response and involves the activation of the innate immune system. It plays a crucial role in many common conditions, such as combating bacterial infections and initiating wound healing. However, when it is not adequately regulated, it can contribute to various diseases and pathological conditions, such as asthma, rheumatoid arthritis, multiple sclerosis, chronic pain, depression, atherosclerosis and heart/vascular disease [89,90,91]. In order to gain a deeper understanding of inflammation, improved methods for its characterization are needed. One such method is the quantitative characterization of inflammation using MPS, as reported by Weaver et al. This involves monitoring the Brownian rotational freedom of MNPs [89]. One of the critical functions of innate immune response and inflammation is phagocytosis. It is the main mechanism of the absorption and clearance of any foreign material, bacteria and dead cells. Another characteristic of inflammation is when the local or global temperature increases as the local circulation and metabolic activity increases to fight invasions. Both phagocytosis and temperature changes impact the Brownian rotational freedom of MNPs, namely the harmonic signals observed [92,93]. The sensitivity of their MPS system is sufficient to measure the MNPs remotely at very low concentrations [94]. Furthermore, the MNP number and relaxation can be independently calculated, allowing for an estimation of the local clearance of MNPs from the volume of interest.

All of these examples have proved that the volumetric-based MPS assay is a wash-free and mix-and-measure approach for rapid and sensitive detection. On the other hand, in comparison to the approaches based on gold/silver nanoparticles in LFAs, a surface-based MPS assay combined with LFA allows for the quantitative detection of biomarkers, as well as the measurements of reaction kinetics [16,95]. In addition, the antigen–antibody binding kinetics in MNP-based biosensors can be accelerated by external fields [95,96,97,98]. All the examples reviewed in this section have been summarized in Table 1.

## 5. MPS for Food Safety

Contaminations of food and crops not only pose significant health risks for consumers but also result in substantial economic losses on a global scale [15,111,112,113,114]. Currently, chromatographic- and immuno-based techniques are employed for identifying foodborne toxins in various sample matrices. However, there is a demand for innovative, high-sensitivity detection technologies that do not require time-consuming procedures or expensive laboratory equipment, yet can still achieve the required detection limit for mycotoxin levels [17]. The United Nations’ Food and Agriculture Organization, along with a study conducted by Eskola et al. in 2019, has indicated that mycotoxins, which are secondary metabolites produced by molds, contaminate approximately 25% of food crops globally [111]. The mycotoxins produced by molds, including Aspergillus, Fusarium and Penicillium species, are highly damaging to human and animal health. These toxins, including aflatoxins, ochratoxins, trichothecenes and zearalenone, are especially harmful. Among these, aflatoxin B1 (AFB1) is the most dangerous, as it is known to cause liver cancer.

Detection technologies that are highly sensitive and reliable are crucial due to the strict regulations and severe effects of mycotoxins. Currently, there are three main analytical technologies used for mycotoxin testing. The most used method is liquid chromatography (LC) coupled with mass spectrometry (MS) in a laboratory setting. Enzyme-linked immunosorbent assays (ELISAs) are also commonly used for highly sensitive mycotoxin testing. Conversely, LFAs are utilized for rapid but less sensitive on-field tests. However, the high cost of equipment, need for highly qualified personnel and possibility of a complex sample cleanup limit the applicability of fast on-site testing. Despite this, LC-MS/MS methods have the significant advantage of high sensitivity and the ability to detect over 500 mycotoxins simultaneously in a single run. In comparison to LC-based methods, ELISA techniques are more cost-effective and faster in the assay procedure, but they also require a laboratory with the appropriate equipment for sample preparation and analysis, according to Renauld and colleagues [115].

An advantage of magnetic biosensors is that the nonmagnetic or paramagnetic matrix effect is not significant. The current laboratory methods for detecting foodborne pathogens from complex food matrices such as milk and meat typically rely on optical labels and involve complex sample preparation procedures [15]. Generally, these methods are time-consuming because they require isolating biomarkers from the sample matrix, removing interfering substances and enriching analytes, which takes several hours at minimum. Moreover, the self-coloration or autofluorescence of samples can contribute to the signal, increase noise and reduce the signal-to-noise ratio [116,117].

**Figure 9:**
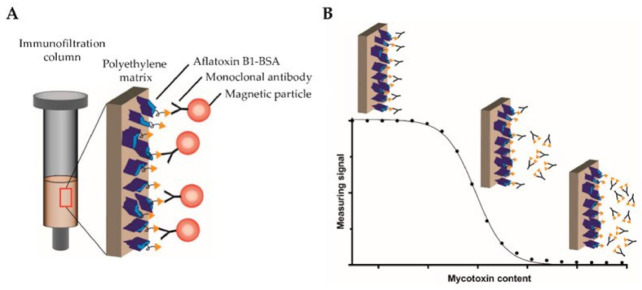
(**A**) An immunofiltration column coated with AFB1-BSA mycotoxin conjugate is used, with biotinylated monoclonal antibodies targeting AFB1 bound to the column. MNPs functionalized with streptavidin are used by MPS to detect the bound antibodies. (**B**) A competitive binding assay method is used. Serially diluted free AFB1 was added in the column to compete for the binding sites with the coated AFB1. Non-saturated antibodies bind to the coated antigen and are retained within the matrix, whereas saturated antibodies are flushed through the column. Then, streptavidin-functionalized MNPs are applied to the column, binding to the retained antibodies, and detected by MPS. Figure reprinted from Ref. [17], distributed under the terms of the Creative Commons Attribution License.

Pietschmann et al. introduced the competitive binding assay on a surface-based MPS platform for the detection of aflatoxin B1 (AFB1), as shown in Figure 9A [17]. Herein, a competitive assay was used since the small molecular structure of AFB1 prevents the simultaneous binding of detection and capture antibodies. In their work, the reaction surface, polyethylene filters, were first coated with AFB1-BSA conjugates (the concentrations of AFB1-BSA were also optimized in their work) and the remaining binding sites were blocked by BSA. Then, the reaction surface was added with biotinylated monoclonal antibodies AFB1_002 for specific binding. After the pre-incubation step, different concentrations of free AFB1 (the test sample) were flushed through the reaction surface, causing the competition of binding sites between the original AFB1 from the reaction surface and the free AFB1 from test sample. A higher concentration of AFB1 from the test sample leads to a higher amount of saturated AFB1_002 antibodies flushed away from the surface. Then, the streptavidin-coated MNPs were added to bind with the remaining AFB1_002 antibodies through a biotin–streptavidin interaction. The MNPs were detected by MPS. Figure 9B depicts the fundamental concept of the MPS-based competitive assay process that occurs in the polyethylene immunofiltration column, along with the associated MPS signal. In order to determine the most effective assay parameters, calibration experiments were conducted to find out the optimal AFB1-BSA coating AFB1_002 antibody concentrations. The experiment utilized free aflatoxin B1 as the competitor, and the dilutions ranged from 0.006 ng/mL to 50,000 ng/mL. It was found that a 0.2 µg/mL AFB1-BSA coating on the reaction surface and 150 ng/mL AFB1_002 antibody combination yielded the highest detection sensitivity and stable data behavior.

**Figure 10:**
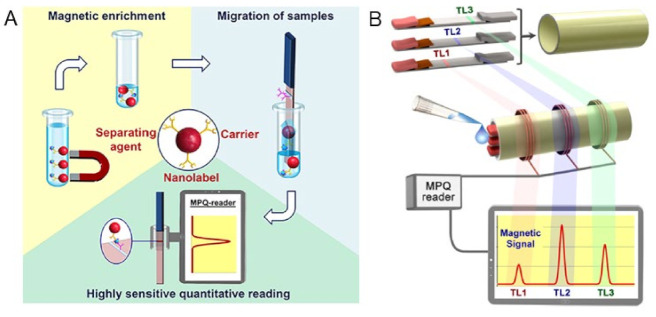
(**A**) An MPS-LFA bioassay platform that utilizes the magnetic separation, magnetic carrier and magnetic signals of MNPs. (**B**) Multiplexed MPS assay utilizing several LF strips. The process involves integrating multiple single-plex LF strips that have different test line positions into a small cartridge (top). Once the sample is deposited onto the cartridge’s front end, it is then inserted into the portable MPQ reader (middle). Finally, the magnetic signals from all of the test strips are read out at the same time (bottom). (**A**) reprinted with permission from Ref. [113], copyright 2019 American Chemical Society. (**B**) reprinted with permission from Ref. [106], copyright 2016 American Chemical Society.

In another work, Bragina et al. reported surface-based MPS combined with LF test strips for the detection of staphylococcal enterotoxin B (SEB) from complex matrices, e.g., in milk, canned meat, baby food and canned mushrooms [113], where the MNPs are used as labels for MPS signal reading, the separating agents are used for target antigen enrichment and carriers are used for the migration of antigens along the LF strip, as shown in Figure 10A. It should be noted that the magnetic separation allows for the sample enrichment on a large volume of liquid and, in addition, makes it possible to process complex matrices. The captured MNPs on the test line of the LF strip is then quantified by their MPS system (called the MPQ reader) to obtain quantitative testing results. They reported a detection limit of 6 pg/mL and a dynamic range of 3.5 orders for detecting SEB from minimal treated food matrices. In contrast to other LFA platforms, which suffer from a decreased sensitivity in complex matrices, this MPS-LFA platform offers a highly sensitive quantitative detection of SEB in food samples with minimal sample preparation. This platform can be further advanced by using multichannel MPQ readers for rapid and multiplexed target analytes screenings. In addition, this detection mechanism allows for assays on large-volume bio-fluids such as urine (up to 100 mL) and blood (up to 10 mL). It can be used in other socially important medical tasks such as the early-stage diagnostics and monitoring of cancer.

In addition, the same group reported the multiplexed assays on this MPS-LFA platform [106]. As shown in Figure 10B, they significantly improved the sensitivity and linear dynamic range of their MPS (MPQ readers) by integrating three measuring inductive coils, which were interrogated by a single processor unit. The advanced electronics enables the reading of these coils separately and sequentially at adjustable time intervals, while still retaining the detection parameters of a 60 zmol sensitivity and 7-order linear dynamic range, which are unmatched by any other detection method. The assays employed a sandwich lateral flow assay (LFA) with MNP labels. In order to conduct the assay, a sample is placed on the sample pad and migrates along the test strip through capillary action. The sample interacts with the dry MNP-Ab conjugate at the conjugation pad, and then the target antigen binds to the MNP-Ab complexes and the capture Ab on the test line (TL). A magnetic control line (CL), like that of conventional LF strips, is added as a control to prevent the false positive result.

## 6. Future Trend of Point-of-Care (POC) Devices

The future of POC devices is set to bring about significant changes in the way that healthcare is delivered. These compact, portable and user-friendly devices are designed to provide rapid and accurate results at the point of care, thereby reducing the need for patients to visit a centralized laboratory. One of the key drivers of the growth in POC devices is the increasing need for cost-effective and efficient healthcare. With the rise of chronic diseases and an aging population, healthcare systems are under significant strain, and POC devices are helping to reduce the burden on these systems. By providing results in real-time, POC devices are enabling healthcare providers to make more informed decisions about treatment and management, reducing the need for repeated visits and saving time and resources. In addition, POC devices are becoming increasingly connected, with many devices now integrating with electronic health records (EHRs) and other healthcare technologies. This integration allows healthcare providers to access results quickly and easily and enables the sharing of patient data between healthcare providers, improving the quality of care and reducing the risk of medical errors.

One of the key challenges facing the growth of POC devices is the need for regulatory approval. Although the devices have been proven to be effective and safe, many still require regulatory approval before they can be widely adopted. However, this is expected to change soon as governments and regulatory bodies become more familiar with the technology and its benefits. Finally, the future of POC devices is also set to be shaped by advances in artificial intelligence (AI) and machine learning (ML). These technologies have the potential to transform the way that POC devices work, enabling them to provide more accurate and personalized results and improving the quality of care. AI and ML are expected to play a significant role in the development of new diagnostic tools, such as predictive models and personalized health assessments. In conclusion, the future of POC devices is set to be exciting, with new and innovative technologies emerging that will transform the way that healthcare is delivered. These devices are helping to reduce the burden on healthcare systems, improve the quality of care and make healthcare more accessible and affordable for patients. With the continued development of POC devices, the future of healthcare is set to be brighter and more efficient than ever before [16,64,82,83,84,85].

The magnetic POC device is still in its infancy stage, with limited products available in the market. Portable magnetic immunoassay platforms require a time-consuming tuning of parameters for use in complex samples such as whole blood and food, but there is no universal method to accelerate this “trial-and-error” stage. Magnetic nanoparticles (MNPs) have great potential to be the leading agents in biosensing due to their unique properties, including stability, ability to serve as solid phases and nontoxicity. Additionally, MNPs can be remotely manipulated by a magnetic field and detected with high sensitivity, even in nontransparent and highly colored samples. MNPs have revolutionized immunoassays by offering a more efficient and rapid enrichment and purification of analytes from complex solutions, as well as reducing matrix effects.

## 7. Conclusions and Outlook

MPS is a rapidly growing field of research that has the potential to revolutionize the way that we detect and diagnose diseases [62,65]. MPS devices are capable of detecting very low concentrations of biomolecules and pathogens, making them an ideal candidate for the early detection and diagnosis of various diseases. In the future, MPS devices are expected to become more compact, affordable and user-friendly. This will make them more accessible to healthcare professionals and researchers, and could lead to a faster, more accurate diagnosis of diseases [98,118].

MPS devices are already being used in several research studies, particularly in the field of molecular biology. For example, researchers are using MPS to study protein–protein interactions and DNA–protein interactions, and to monitor the progress of enzymatic reactions [119,120]. As the technology continues to develop, it is expected that MPS devices will become more widely used in other areas of research, such as drug discovery and development. With the ability to rapidly and accurately screen thousands of molecules, MPS devices could greatly accelerate the drug discovery process, leading to a faster development of new drugs [121].

One of the most exciting prospects for MPS devices is in the field of personalized medicine. With the ability to detect very low concentrations of biomolecules, MPS devices could enable an earlier diagnosis of diseases and allow for more targeted and personalized treatment plans. By monitoring the progress of treatment, healthcare professionals could adjust the treatment plan as needed, leading to better outcomes for patients [2,122]. As the technology continues to develop and become more affordable, it is expected that MPS devices will become more widely used in clinical settings, enabling more personalized and targeted treatment plans for patients.

In conclusion, the development of MPS devices has enabled the sensitive and rapid detection of various analytes in complex biological samples. With their ability to amplify magnetic signals, MPS devices have shown great potential in advancing the field of diagnostics, including the detection of infectious diseases, toxins and cancer biomarkers [111,123]. The use of MPS devices in point-of-care testing and resource-limited settings can greatly improve access to rapid and reliable diagnostic tools, thereby enhancing disease management and patient outcomes.

In the future, developing affordable testing kits and low-cost MNPs are important challenges for using MPS in POC applications. The magnetic properties of MNPs, such as their magnetic response and relaxation time, are unique and depend on various factors, such as their size, shape and saturation magnetization [8]. Each type of MNP has a unique MPS spectrum that can be analyzed to distinguish between them based on their harmonic amplitudes and phases [124]. By conjugating different capture probes such as antibodies, DNA, RNA and peptides onto different types of MNPs, it is possible to label different target analytes specifically with different types of MNPs. This property of MNPs has great potential in MNP-based cell labeling and magnetic flow cytometry [125]. Unlike traditional fluorescent flow cytometers that rely on fluorescent markers for cell labeling, magnetic labels (i.e., different types of MNPs) could lead to the development of magnetic flow cytometers. The unique magnetic response of each type of MNP also enables the design of MPS-based multiplexed bioassays [124,126,127]. Multiplexed assays and labeling in magnetic flow cytometry require improvements in MNP synthesis methods to provide nanoparticles with higher saturation magnetizations and better size uniformity. Furthermore, advanced MNP surface biofunctionalization technologies are necessary to increase shelf life and biocompatibility [53].

Moving forward, further research and development in MPS technology can lead to the creation of more advanced and user-friendly devices with an increased sensitivity and specificity. Additionally, the integration of MPS devices with other diagnostic tools, such as microfluidic systems and smartphone technology, can further enhance the capabilities and accessibility of MPS devices. Overall, the future of MPS devices in academia holds great promise in advancing the field of diagnostics and improving healthcare outcomes [128].

## Figures and Tables

**Table 1:** Summary of MPS-based biomedical applications.

MPS Platform	MNP	Target Analyte	Matrices	Detection Range	Detection Limit	Assay Time	Drive Field	Ref.
Volumetric-based	30 nm single-core	SARS-CoV-2 spike protein	Buffer	—	1.56 nM	—	Dual-frequency	[99]
	30 nm single-core	H1N1 nucleoprotein	Buffer	—	44 nM	10 s	Dual-frequency	[88]
	70 nm multi-core	SARS-CoV-2-specific antibody	Serum	—	—	21 min	Dual-frequency	[86]
	100 nm multi-core	Inflammation and infection	—	—	—	—	Mono-frequency	[89]
	35 nm single-core	Goat anti-human IgG	—	—	0.5 mg/mL (3.1 mM)	—	Dual-frequency	[100]
	50 nm multi-core	Streptavidin	—	150 pM–1200 mM	50 pM	—	Mono-frequency	[87]
	50 nm multi-core	Thrombin	Buffer	4–20 nM	4 nM	10s	Mono-frequency	[87]
	50 nm multi-core	ssDNA	Buffer	200–2000 pM	100 pM	—	Mono-frequency	[87]
	100 nm multi-core	Mouse granzyme B	Buffer	—	10 pM	—	Dual-frequency	[101]
	50 nm multi-core	Blood clot	—	—	—	—	Dual-frequency	[102]
	100 nm multi-core	Blood clot	—	—	—	—	One DC field added on top of one mono-frequency field	[103]
Surface-based	75 nm multi-core	Cholera toxin	Water	0.2 ng/mL–700 ng/mL (12 nM–438 mM)	0.2 ng/mL (12 nM)	—	Dual-frequency	[104]
	0.5–1 μm multi-core	C-reactive protein (CRP)	Saliva, urine and blood serum	25 ng/mL–2.5 μg/mL (156 nM–15.6 μM)		11.5 min	Dual-frequency	[105]
	196 nm multi-core	Prostate specific antigen (PSA)	Serum	—	25 pg/mL (156 pM)	30 min	Dual-frequency	[64]
	198 nm multi-core	Botulinum neurotoxins A, B and E	Buffer Milk Apple juice Orange juice	—	185, 140, 350 pg/mL (1159, 876, 2191 pM) 197, 143, 254 pg/mL (1233, 895, 1590 pM) 307, 142, 465 pg/mL (1922, 870, 2567 pM) 287, 139, 410 pg/mL (1797, 870, 2567 pM)	—	Dual-frequency	[106]
	0.5–1 μm multi-core	*Yersinia pestis* antigen F1	Buffer and blood	25–300 ng/mL (156–1870 nM)	2.5 ng/mL 15.6 nM	—	Dual-frequency	[107]
	200 nm multi-core	Potato virus X (PVX)	Buffer	20 μg/mL 120 μM	56 ng/mL 350 nM	30 min	Dual-frequency	[108]
	0.5–1 μm multi-core	*Francisella tularensis lipopolysaccharide*	Buffer and rabbit serum	10^4^–10^6^ cfu/mL	—	—	Dual-frequency	[109]
	700 nm multi-core	Aflatoxin B1	Aflatoxin B1	—	—	4.5 h	Dual-frequency	[17]
	198 nm multi-core	Free thyroxine (fT4)	Serum	0.01–10 pM	16 fg/mL (20 fM)	30 min	Dual-frequency	[110]

## Data Availability

Not applicable.
